# Prognostic factors for return to work and work disability among colorectal cancer survivors; A systematic review

**DOI:** 10.1371/journal.pone.0200720

**Published:** 2018-08-15

**Authors:** Chantal M. den Bakker, Johannes R. Anema, AnneClaire G. N. M. Zaman, Henrika C. W. de Vet, Linda Sharp, Eva Angenete, Marco E. Allaix, Rene H. J. Otten, Judith A. F. Huirne, Hendrik J. Bonjer, Angela G. E. M. de Boer, Frederieke G. Schaafsma

**Affiliations:** 1 Department of Occupational and Public Health, VU University medical center, Amsterdam Public Health research institute, Amsterdam, The Netherlands; 2 Department of Surgery, VU University medical center, Amsterdam, The Netherlands; 3 Academic Medical Center, Amsterdam Public Health research institute, Coronel Institute of Occupational Health, University of Amsterdam, Amsterdam, The Netherlands; 4 Department of Epidemiology and Biostatistics, Amsterdam Public Health research institute, VU University medical center, Amsterdam, The Netherlands; 5 Institute of Health & Society, Newcastle University, Newcastle, United Kingdom; 6 Department of Surgery, Institute of Clinical Sciences, Sahlgrenska Academy, Gothenburg University, Göteburg, Sweden; 7 Department of Surgical Sciences, University of Torino, Torino, Italy; 8 Medical Library, Vrije Universiteit, Amsterdam, The Netherlands; 9 Department of Gynaecology, VU University medical center, Amsterdam, The Netherlands; University of South Alabama Mitchell Cancer Institute, UNITED STATES

## Abstract

**Background:**

Colorectal cancer is diagnosed progressively in employed patients due to screening programs and increasing retirement age. The objective of this study was to identify prognostic factors for return to work and work disability in patients with colorectal cancer.

**Methods:**

The research protocol was published at PROSPERO with registration number CRD42017049757. A systematic review of cohort and case-control studies in colorectal cancer patients above 18 years, who were employed when diagnosed, and who had a surgical resection with curative intent were included. The primary outcome was return to work or work disability. Potentially prognostic factors were included in the analysis if they were measured in at least three studies. Risk of bias was assessed according to the QUality In Prognosis Studies tool. A qualitative synthesis analysis was performed due to heterogeneity between studies. Quality of evidence was evaluated according to Grading of Recommendation Assessment, Development and Evaluation.

**Results:**

Eight studies were included with a follow-up period of 26 up to 520 weeks. (Neo)adjuvant therapy, higher age, and more comorbidities had a significant negative influence on return to work. A previous period of unemployment, extensive surgical resection and postoperative complications significantly increased the risk of work disability. The quality of evidence for these prognostic factors was considered very low to moderate.

**Conclusion:**

Health care professionals need to be aware of these prognostic factors to select patients eligible for timely intensified rehabilitation in order to optimize the return to work process and prevent work disability.

## Introduction

Colorectal cancer is the third most common type of cancer globally in men and the second in women [1]. As a result of improvements in cancer treatment and general healthcare the average 5-year relative survival worldwide of colon cancer is now 57% and of rectal cancer 56% [2]. The lifetime risk of developing colorectal cancer in many regions worldwide is around 5% [3]. Over the past two decades, the number of colorectal cancer screening modalities has increased and many population-based programs have been implemented [4]. Currently, most developed countries already have some form of screening in place. As a result of screening, colorectal cancer will be discovered and treated at an earlier stage [5]. The number of colorectal cancer survivors is expected to increase further due to an ageing population in developed countries, rising survival rates and the availability of screening.

Short-term morbidity and mortality are most commonly used endpoints of colorectal cancer treatment [6–7]. In contrast, there is limited literature available on long term post-operative recovery and rehabilitation of colorectal cancer patients. Recovery or rehabilitation has been defined as the total or full recovery of a sick or disabled person by therapeutic measures and return to activities of daily living within the limitations of the person’s physical disability [8]. The time to full recovery after major abdominal surgery is currently not determined, however there are clear signs that a prolonged recovery period may be associated with a compromised quality of life and depression, as well as shorter survival and severe economic burden for patients as well as for society [9–10].

A critical element for full recovery after surgery is return to normal activities of which return to work is considered one of the most important endpoint. Being able to work is seen as a significant milestone of full recovery by many cancer patients [11]. It gives them self-confidence, social interactions, a feeling of recovery and financial security [11–12]. At the moment more than 30% of colorectal cancer survivors are below 65 years and are therefore often still active in the workforce [1,3,13]. With the increasing retirement age in many developed countries, it is expected that more people will be diagnosed with colorectal cancer while they are an active part of the workforce [14]. This increasing number of colorectal cancer patients in the overall working population will have a profound economic impact in terms of lost productivity due to temporary work cessation, permanent departure from the workforce (temporary reduction of working hours or workforce departure due to work disability) and premature mortality [5,15].

Information about factors which may positively or negatively influence return to work or work disability enables health care professionals to provide better information about vocational rehabilitation to patients and their families. Therefore, the aim of this systematic review was to give an overview of potentially relevant prognostic factors for the primary outcome return to work or work disability of colorectal cancer survivors.

## Methods

A systematic review was performed following the ‘Preferred Reporting Items for Systematic reviews and Meta-Analyses’ (PRISMA) guidelines [16]. A research protocol for this review was agreed upon by all co-authors before starting the literature searches. The research protocol was published online at the PROSPERO International prospective register of systematic reviews (http://www.crd.york.ac.uk/PROSPERO/) under registration number: CRD42017049757.

### Eligibility criteria

Studies fulfilling the following inclusion criteria were included:

#### I. Study designs

Retrospective- and prospective cohort studies as well as studies with a case control design were included. There was no limitation to the minimal length of the follow-up period in the cohort studies.

#### II. Participants

Studies on patients diagnosed with colorectal cancer of 18 years and older, who were working at time of diagnosis and who had a surgical resection with a curative intent were considered eligible. Studies investigating multiple cancer diagnoses were only included when separate results were reported for colorectal cancer patients.

#### III. Outcome measures

The primary outcome of this study was return to work or work disability. Return to work was defined as having (fully or partially) returned to work in previous or equal work after a period of sick leave during or at a certain follow up measurement (e.g. after 1 year). Work disability was defined as not being able to meet the demands of gainful activity during or at a certain follow up measurement, due to functional limitations caused by impairment. Work disability was considered as a temporary or irreversible form of not working e.g. outcome measures such as: disability pension, sickness absence, work cessation, work disability or incapacity were included [17–18].

#### IV. Prognostic factors

Prognostic factors concerning 1. person-related (e.g. age, gender); 2. diagnosis- or treatment-related (e.g. (neo)adjuvant therapy, type of surgery); and 3. occupational-related factors (e.g. type of work (blue/white collar) and workload) were eligible. If articles reported on the same study cohort, initially the index article was included in this review; if the other article reported on additional prognostic factors, these factors were also included.

### Search methods for identification of studies

The search strategy was developed with assistance from an experienced clinical librarian (RO) to ensure an optimal search. The following electronic databases were used: (I) The Cochrane Library, (II) Ovid MEDLINE, (III) Ovid EMBASE, (IV) PsycINFO (EBSCO host) and (V) Cumulative Index to Nursing and Allied Health Literature (CINAHL) (EBSCO host). Additionally, the database of prognostic studies maintained by the Cochrane Prognosis Methods Group (PMG) was used. References of papers considered eligible were cross-checked to identify any further articles. Search terms included controlled terms (MeSH in PubMed and Emtree in Embase) as well as free text terms. Only free text terms were used in The Cochrane Library. Search terms expressing ‘return to work’ were used in combination with search terms comprising ‘colorectal cancer’. Studies until 16 May 2018 were included. Only articles in English or Dutch were eligible. The full electronic search strategy for MEDLINE is shown in S1 File. Duplicate articles were excluded.

### Study selection

Studies were selected independently by two of the authors (CdB and FS). Initially, the titles and abstracts were screened and full reports from potentially relevant studies were retrieved. The authors used EndNote to assess and document the full reports on inclusion or exclusion according to the predefined selection criteria. Disagreements were resolved by discussion and where agreement could not be reached, a third reviewer was consulted (AdB).

### Data extraction

Data extraction was performed by CdB and checked by FS. Data on author, year of publication, setting, study population, study design, follow-up duration, measuring methods, timing of outcome assessment, and prognostic factors were extracted. The odds ratio, hazard ratio, risk ratio, incidence rate ratio or regression coefficient was extracted as the estimate of the effect size. Univariate effect sizes were used even if the multivariate effect sizes were also presented, as we were interested in prediction and not to assess causality [19]. Disagreements were resolved by discussion or by involving JA as arbiter. When there were uncertainties about the reported data, authors of included studies were contacted. The authors of Van den Brink et al 2005, Gordon et al 2014 and Carlsen et al 2013 were all contacted, but only Van den Brink et al. replied but they could not give more clarity about their data. As a result, for all studies only the published data was used in this review.

### Quality assessment of individual studies

For assessing the quality of individual studies the widely used QUality In Prognosis Studies tool was applied [20–21]. Six domains are critical for assessing biases that potentially distort the findings of prognosis research: (I) selection of study participants, (II) study attrition, (III) prognostic factor measurement, (IV) outcome measurement, (V) study confounding and (VI) statistical analysis and reporting. For each of these 6 domains, the responses ‘yes’, ‘partial’, ‘no’ or ‘unsure’ for three up to seven items within each domain are combined to assess the risk of bias. An overall rating for each domain is assigned as ‘high’, ‘moderate’ or ‘low’ risk of bias. The QUality In Prognosis Studies assessment for each study was independently completed by CdB and AZ. Differences were resolved by discussion or by referral to FS. A study was considered to be of low risk of bias when the items were rated as low or moderate on all of the six domains, with at least four rated as low (of which the outcome measurement domain must be rated as low at least). A study was scored as high risk of bias if two or more of the domains were scored as high. The remaining studies were scored as moderate [21–22].

### Data analysis

It was decided to include a potential prognostic factor in the analysis when this factor was measured in at least three different studies. This threshold was chosen to increase the ability to draw conclusions about the consistency and relevance of these factors [19,23]. After data extraction and selection of prognostic factors the homogeneity between included studies per prognostic factor was assessed. A meta-analysis of prognostic factors was considered inappropriate due to the high heterogeneity in the definition and/or operationalization of the prognostic factors between the studies. To have more insight into the effects per factor on the outcome measures, a forest plot (without the pooled effect) was used. For these plots, the reported effect parameters and 95% confidence intervals in individual studies of prognostic factors were first converted into effect sizes that measured the effect comparably to ensure comparison of each prognostic factor. Regression coefficients were converted into effect sizes using the standard deviation of the prognostic factor, and odds ratios were converted into risk ratios using the non-exposed prevalence. For the analysis the number of studies evaluating a specific prognostic factor and the consistency of the direction of the results of these studies was taken into account. Although, the follow-up periods differed across included studies, the directions of the effect from the prognostic factors on our primary outcomes were comparable. As such, we did not further stratify the analysis based on the follow-up period. A potentially prognostic factor was considered consistent if >75% of the studies reporting on this factor showed the same statistically significant direction of the association with the outcome. After initial review, an exception to this criterion was applied in case of three studies. In that case, it was decided to assume that two out of three studies (i.e. 67%) had to show statistically significant results in the same direction. Prognostic factors with a significant association in <75% of the included studies were considered inconclusive [24–25].

### Grading of Recommendation Assessment, Development and Evaluation

The Grading of Recommendation Assessment, Development and Evaluation on prognosis research was used to rate the overall evidence per factor in order to evaluate the limitations of all eligible studies [26]. The Grading of Recommendation Assessment, Development and Evaluation was assessed according to the standard framework. Evidence on prognostic studies was evaluated by six factors that may decrease quality: (I) phase of investigation; (II) study limitations; (III) inconsistency; (IV) indirectness; (V) imprecision; and (VI) publication bias. Factors that may improve the quality of evidence were; (I) moderate or large effect size; and (II) exposure-response gradient.

## Results

**Fig 1:**
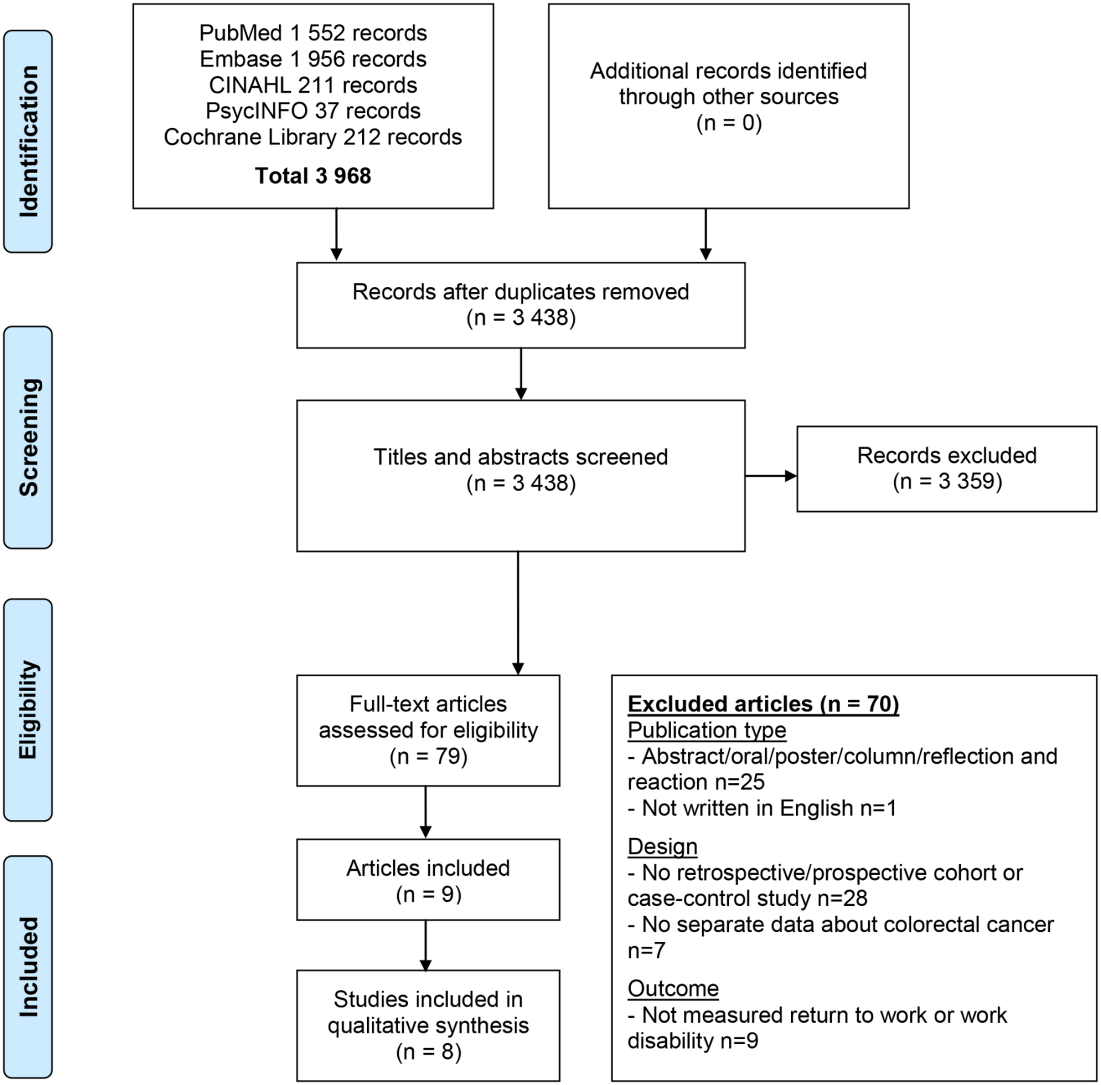
PRISMA diagram showing selection of studies for systematic review.

The literature search resulted in a total number of 3 968 hits. After duplicate removal, 3 438 hits were screened on title and abstract. This resulted in 79 full-text articles that were assessed for eligibility, of which eight studies described in nine articles met the inclusion criteria (Fig 1).

### Study characteristics

The characteristics of the included studies are presented in Table 1 [27–35]. Two of the included studies focussed only on return to work [28–30], four studies on work disability [32–35], and two studies on both outcomes [27–31]. Variations in definitions and measurements of work disability were evident. Three studies reported about disability pension, two about work cessation and one about sickness absence. Of the eight included studies, most studies (n = 6 studies) were prospective cohort studies [27–31,33–34]. The remaining studies were case-control studies [32,35]. Studies were executed in six different countries; most of them in Europe (n = 6 studies) [29–33,35], and two in Australia [27–28,34]. There was considerable variation across studies regarding sample size and length of follow-up. The sample size ranged from 50 up to 4343 patients. The follow-up period ranged from 26 up to 535 weeks; five of the included studies had a follow-up longer than 1 year [29,31–33,35]. For return to work, the articles of Gordon et al 2014 and Lynch et al 2015 dealt with one study cohort and were therefore combined to one study for this outcome measure [27–28]. The study by Van den Brink et al 2005 reported only regression coefficients without SD’s, making it impossible to calculate effect sizes. However, with these regression coefficients a positive or negative direction of the effect on return to work could be determined. The effects of Van den Brink et al 2005 are therefore reported in the plots with an asterisk (*) [29].

**Table 1:** Characteristics of included articles on return to work or work disability in colorectal cancer survivors.

Characteristics of included articles										
Author	Year	Country	Design	n	Age in years mean (sd)	Gender male (%)	Follow-up (week)	Measurement	Outcome	Operationalization of the outcome
**Articles discussing Return to Work**										
Bains et al.	2011	United Kingdom	Prospective cohort study	50	52.5 (5.4)	28 (56)	26	Questionnaire	Employment	Working vs not working
van den Brink et al.	2005	The Netherlands	Nested cohort study	292	52 (7)	144 (49.3)	104	Database questionnaire	Paid labor resumption	Working vs not working
**Articles discussing Work Disability**										
Chen et al.	2015	Sweden	Matched cohort study	2 815	55 (NA)	1 686 (59.9)	520	Database National register	Disability pension	DP cases per person-years at risk
Chen et al.	2016	Sweden	Prospective cohort study	3 438	56 (20–61) median (range)	1 985 (57.7)	260	Database National register	Sick leave	Net days of SL and DP
									Disability pension	
Gordon et al.	2008	Australia	Population-based longitudinal study	975	60.2 (10.4)	621 (63.7)	52	Database questionnaire	Work cessation	Yes or no
Hauglann et al.	2014	Norway	Controlled cohort study	648	51 (NA)	381 (58.8)	728	Database National register	Disability pension	Yes or no
**Articles discussing both Return to Work and Work Disability**										
Carlsen et al.	2013	Denmark	Register-based cohort study	4 343	53.8 (NA)	2430 (56.0)	535	Database National register	Sickness absence	Yes or no
									Return to work	Working vs not working
Gordon et al. / Lynch et al	2014 / 2016	Australia	Prospective population-based study	239	56 (5.5)	160 (67)	52	Database & questionnaire	Work cessation	Not working at 12 months
									Work resumption	Net days of RTW

SL = Sick leave; DP = Disability pension; RTW = Return to work

### Risk of bias within studies

**Fig 2:**
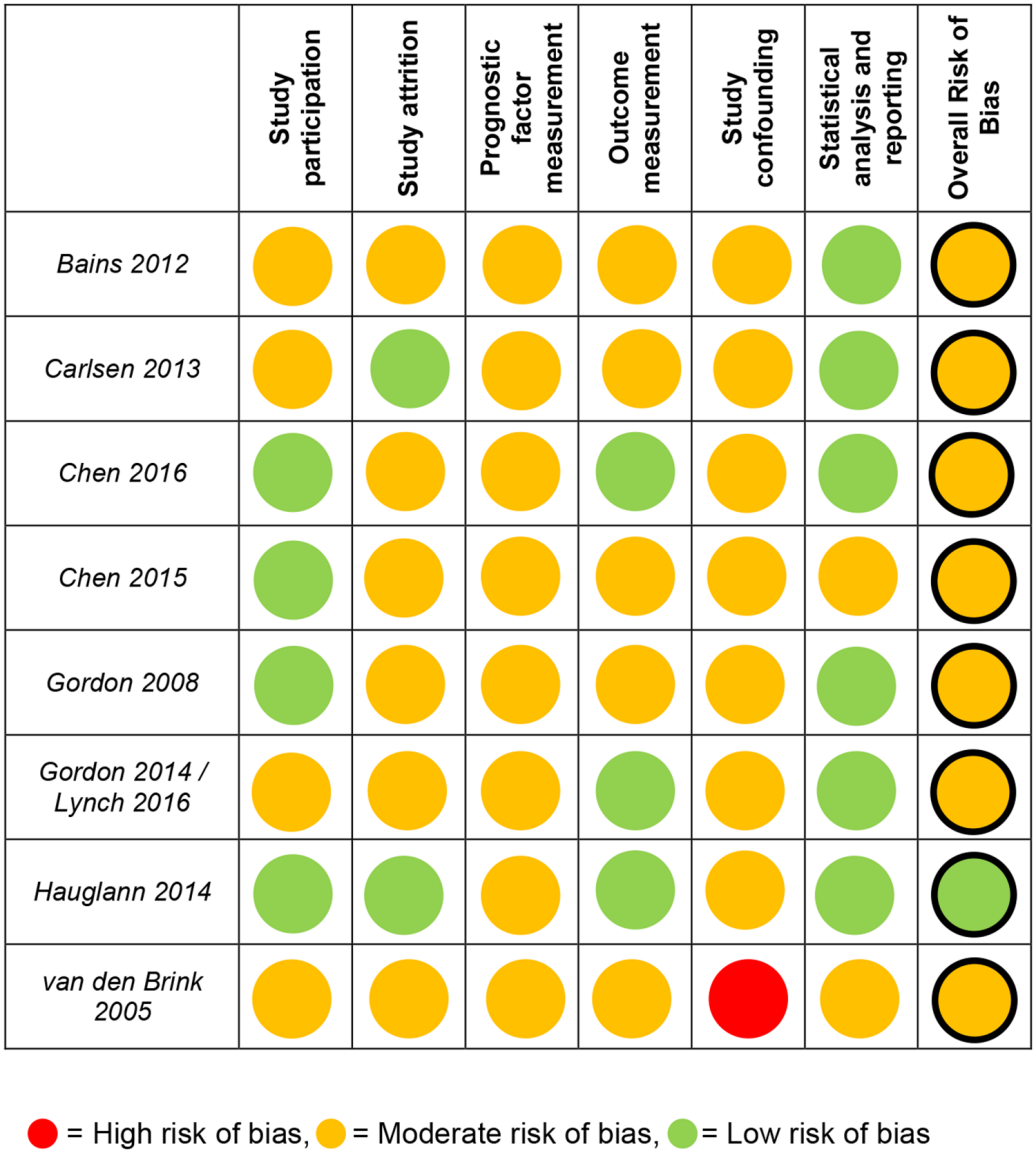
Risk of bias according to the QUIPS tool. Red circle = High risk of bias, orange circle = moderate risk of bias, green circle = low risk of bias.

The overall ‘Risk of Bias’ of each included study is presented in Fig 2. Overall agreement on methodological quality scores between the reviewers was 81.5%. Cases where reviewers disagreed mainly concerned the rating of attrition of patients and the confounding factors. Consultation of a third reviewer was necessary to resolve disagreement for 18.5% of all scores. One study was considered to have low risk of bias, all other seven studies have moderate risk of bias mainly due to the variety in measuring and categorization of the prognostic factors.

### Included prognostic factors

In Table 2, all reported prognostic factors are presented. The amount and type of potentially prognostic factors investigated varied per study. The operationalization of these factors also differed between studies. Four studies assessed a total of 32 potential prognostic factors for return to work [27–31] and six studies assessed a total of 33 potential prognostic factors for work disability [27,31–35]. In total 10 factors on person-related factors, 14 factors on diagnosis- or treatment-related factors and 8 factors on occupational-related factors in the return to work studies were measured. Furthermore, in total 9 factors on person-related factors, 14 factors on diagnosis- or treatment-related factors and 10 factors on occupational-related factors in the work disability studies were measured. The prognostic factors that were analysed in at least three studies are shown in bold in Table 2.

**Table 2:** Prognostic factors measured in included articles.

Prognostic factors	
Return to Work	Work Disability
***Person-related***	***Person-related***
**Age**	**Age**
**Education**	**Gender**
Gender	**Education**
Vegetable/fruit consumption	BMI
Alcohol consumption	Residence area
Smoking status	Marital status
Sitting time	Private health insurance
Marital status	Children in household
BMI	People in household
Perceived prosperity	
***Diagnosis- or treatment-related***	***Diagnosis- or treatment-related***
**Comorbidity**	**Type of surgery**
**(Neo)adjuvant therapy**	**Postoperative complications**
Type of surgery	**(Neo)adjuvant therapy**
Type of cancer	**Stage**
Stage	Type of cancer
ASA classification	ASA classification
Curative operation	Curative operation
Postoperative complications	Reoperation
Stoma fitted	Hospital volume
Hospital length of stay	Energy
Phyiscal Symptom Distress	Physical component of SF-12
Limitations in daily activities	Surgical complications
Energy	Non-surgical complications
Physical activity	Comorbidities
***Occupational-related***	***Occupational-related***
**Occupation**	**Occupation**
Income	**Previous unemployment**
Previous periods of work	Income
Previous period of sick absence	Previous periods of work
Previous unemployment	Previous period of sick absence
Job self-efficacy	Employer size
Work ability	Time at employer
Employer size	Work contract
	Work hours prior to cancer
	Total household income

Bolded and underlined prognostic factors are measured in at least 3 studies and thus included in the analysis.

### Prognostic factors for return to work

**Fig 3:**
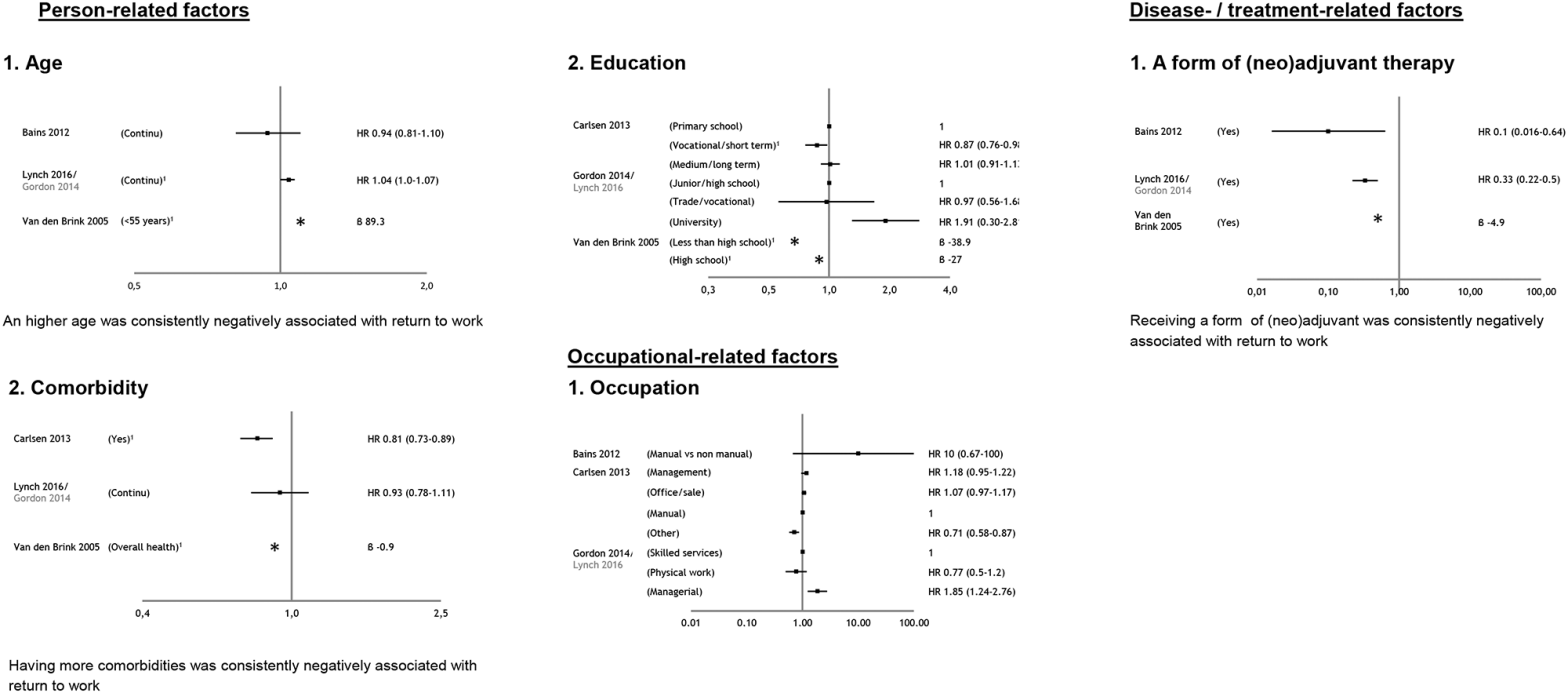
Plots per prognostic factor, measured in at least 3 studies, for return to work (RTW). 1 Significant different. * direction of regression coefficient. Gordon is parent study, Lynch other study (both using the same study cohort).

In Fig 3 the prognostic factors for return to work are presented. In total five factors were included in this analysis, two person-related-, two diagnosis- or treatment-related-, and one occupational-related factor based on the criterion that a factor should be measured in at least 3 studies. The effect sizes of non-included factors on return to work are presented in S1 Table. Of the remaining 27 potential prognostic factors, four factors were investigated in two studies and 23 only in one study.

#### Person-related factors

Three studies reported on *age* [28–29,33], and three on *education* [27,29,31]. An higher age was consistently negatively associated with return to work. For the factor education inconclusive evidence was found for their association with return to work, because of an opposite effect in these studies (Fig 3).

#### Diagnosis- or treatment-related factors

Three studies reported on *(neo)adjuvant therapy* (supplementary to surgery) [28–30], and three on *comorbidity* [28–29,31]. Receiving (neo)adjuvant therapy and having more comorbidities were consistently negatively associated with return to work (Fig 3).

#### Occupational-related factors

Three studies reported on type of *occupation* (manual vs non-manual work was investigated) [27,30–31]. For this factor inconclusive evidence was found on return to work as in none of the studies a significant effect on return to work was found (Fig 3).

### Prognostic factors for work disability

**Fig 4:**
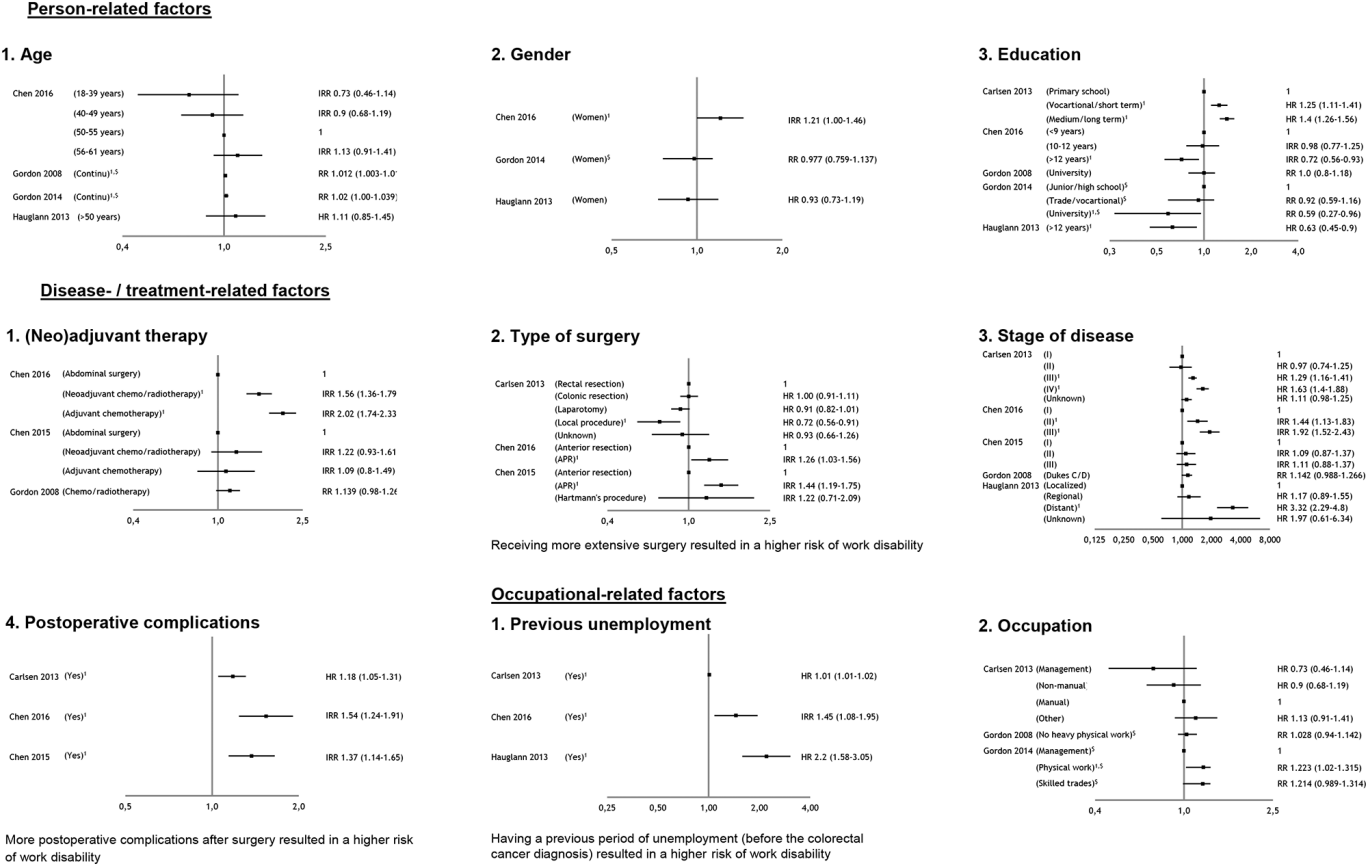
Plots per prognostic factor, measured in at least 3 studies, for work disability. 1 Significant different. $ Converted from OR into RR.

In Fig 4 the prognostic factors for work disability are presented. In total, nine factors were included in this analysis, three person-related-, four diagnosis- or treatment-related- and two occupational-related factors based on the criterion that a factor should be measured in at least three studies. The effect sizes of non-included factors on return to work are presented in S2 Table. Of the remaining 24 potential prognostic factors, five factors were investigated in two studies and 19 only in one study.

#### Person-related factors

Four studies reported on *age* [27,33–35], three on *gender* [27,33,35], and five on *education* [27,31,33–35]. For all these factors inconclusive evidence was found for their association with work disability. In two out of four studies a higher age had a significant effect on the risk for work disability. For gender in only one out of the three studies a significant risk for work disability was found for women compared to men. And for the factor education, three studies reported a significant risk for work disability due to lower type of education, however other studies reported no or an opposite effect (Fig 4).

#### Diagnosis- or treatment-related factors

Three studies reported on *(neo)adjuvant therapy* (supplementary to surgery) [32–34], three studies on *type of surgery* [31–33], five studies on *stage of* disease [31–35], and three studies on *postoperative complications* [31–33]. Receiving more extensive surgery and experiencing more postoperative complications after surgery resulted in a higher risk of work disability. For the factors *stage of disease* and *(neo)adjuvant therapy* (supplementary to surgery) inconclusive evidence was found on work disability. For *disease stage* in only three out of five studies a significant increase in the risk for work disability was reported. For (*neo)adjuvant therapy* (supplementary to surgery) in only one out of three studies a significant increase in the risk for work disability was reported (Fig 4).

#### Occupational-related factors

Three studies reported on *previous unemployment* [31,33,35], and three on *occupation* [27,31,34]. Having a previous period of unemployment (before the colorectal cancer diagnosis) resulted in a higher risk of work disability. For the factor *occupation* (manual vs non-manual work was investigated) inconclusive evidence was found on the risk for work disability, because only one out of three studies reported this effect (Fig 4).

### Grading of Recommendation Assessment, Development and Evaluation

The Grading of Recommendation Assessment, Development and Evaluation classification per prognostic factor for return to work and work disability is presented in Table 3. Moderate evidence was found for the factor *postoperative complications* according to this classification system, only downgrading for study limitations was necessary given the moderate risk of bias of included studies.

**Table 3:** Grading of Recommendation Assessment, Development and Evaluation classification per significant prognostic factor for return to work and work disability.

	*GRADE factors*								Overall quality
	Phase of investigation	Study limitations	Inconsis-tency	Indirectness	Imprecision	Publication bias	Moderate / large effect size	Dose effect	
Return to work									
*Age*	√	✘	✘	✘	√	√	NA	✘	+
*(Neo)adjuvant therapy*	√	✘	√	√	✘	√	NA	✘	+ +
*Comorbidites*	√	✘	✘	✘	√	√	NA	✘	+
Work disability									
*Type of surgery*	√	✘	√	✘	√	√	NA	✘	+ +
*Postoperative complications*	√	✘	√	√	√	√	NA	✘	+ + +
*Previous unemployment*	✘	✘	√	√	√	√	NA	✘	+ +

Grading of Recommendation Assessment, Development and Evaluation factors:

√ = no serious limitations

✘ = serious limitiations

NA = not applicable or unknown

For overall quality of evidence:

+ = very low

++ = low

+++ = moderate.

Low evidence was found for *(neo)adjuvant therapy*, *type of surgery* and *previous unemployment*. All these factors were downgraded for study limitations due to the moderate risk of bias of included studies. The factor *(neo)adjuvant therapy* was downgraded for imprecision given the included underpowered study of Bains et al which did not provide a rationale for the chosen sample size [30]. The factor *type of surgery* was downgraded for indirectness due to the variation in operationalization of the prognostic factor itself. The factor *previous unemployment* was downgraded for phase of investigation due to included studies that were not primarily designed to examine prognostic factors for work disability, but this was performed as sub analysis.

Very low evidence was found for *age* and *comorbidities*. These factors were downgraded for study limitations due to moderate risk of bias of included studies, for inconsistency because only two out of the three studies found a significant effect, and for indirectness due to the variation in operationalization of the prognostic factor itself.

## Discussion

*A form of (neo)adjuvant therapy*, *higher age and more comorbidities* had a negative influence on return to work. For the other two included factors on return to work inconclusive results were found. *A previous period of unemployment*, *extensive surgery* and *postoperative complications* were considered to increase the risk for work disability. For the other six included factors on work disability inconclusive results were found.

For this review, only a limited number of studies on prognostic factors on colorectal cancer survivors’ return to work and/or work disability were available. This can probably be explained by the high average age at diagnosis in the past, as such patients were typically no longer part of the work force. Another, notable finding was the various ways of measuring the outcome measures, mostly depending on the nation in which the study was executed. The definition of return to work and work disability is not consistent across national social security systems or other stakeholders responsible for the financial benefits for sick workers, explaining the reported variation of these outcome measures [36–38]. Partly as a consequence, there is also no consensus in the research community how these outcomes and prognostic factors should be measured or operationalized.

Prognostic factors can be divided into non-modifiable and potentially modifiable factors. The relevant prognostic factors for return to work and work disability measured in this review were non-modifiable (diagnosis / treatment) or less easy to modify (therapy-related). However, the assessment of these non- or difficult modifiable factors remains relevant information for health care professionals advising about return to work / work disability for colorectal cancer survivors [39]. Early identification of risk factors will improve the guidance for return to work [39]. A number of systematic reviews regarding multiple cancer diagnoses have also investigated potential prognostic factors for return to work [40–49]. Diagnosis- or treatment-related factors ((neo)adjuvant therapy, the type of surgery or postoperative complications) were also relevant prognostic factors in these systematic reviews [40,46,48]. However, these reviews also reported on more relevant occupational-related factors, such as blue vs white collar professions and the amount of working hours compared to the results of this review [40–49]. This may be explained by the limited number of included studies in our review. Identification of work related factors is however valuable when interpreting the outcome of return to work or work disability after colorectal cancer treatment. Besides, these factors are usually more modifiable and as such can be used to facilitate return to work (e.g. adjusting manual into non-manual work, optimizing relationships with colleagues and employers) [5].

The main strength of this review is that this is the first systematic review regarding prognostic factors for return to work and work disability among colorectal cancer survivors. This review has revealed that much more longitudinal observational studies are necessary focusing on particular relevant factors for this target group that will increase in the number of people that wishes to return to work after treatment over the next few years. Another strength of this review is the methodological quality, ensured by following the PRISMA guidelines for systematic reviews [16]. An extensive literature search was conducted based on a comprehensive search strategy developed by an clinical librarian with expertise in the field. By including both cohort studies as well as case control studies, we are confident that this review presents a full overview of existing studies on this topic.

Despite the extensive search, a potential limitation may be the exclusion of non-English studies and grey literature. Furthermore, all studies investigating multiple cancer diagnoses were excluded when no separate results were reported for colorectal cancer survivors, this may have caused some bias in our results. In addition, the majority of the studies did not make a distinction between the diagnoses colon- or rectal cancer with corresponding different treatment strategies. As a result we could not separate these diagnoses in the analysis. Another limitation can be that the differences in categorizations of prognostic factors may be of influence on the total conclusion of a prognostic factor. It was decided to draw conclusions despite these differences. In addition, our threshold of only reporting on a factor that was included in at least three studies may have been too strict. Hereby, it is possible that other important factors may not be included in the analysis. In order to give full disclosure for all prognostic factors, the categorization is presented in the plots in Figs 3 and 4 and the number of studies investigating a prognostic factor are presented in S1 and S2 Tables. Lastly, the QUIPS tool is a non-validated instrument which could give room for personal interpretation. This was addressed by discussing the use of this tool in advance with an expert (HdV) in the prognostic research field. In addition, the QUIPS tool is recommended by the Cochrane Methods Prognosis group and designed for prognosis studies addressing all common sources of bias [50]. Based on this, we considered the tool as suitable for evaluating risk of bias. The allocated quality marks that were used to discriminate study quality as well as the chosen cut-off points are considered arbitrary.

The primary postoperative focus of most health care professionals (e.g. surgeons and / or oncologists) is naturally on the patients’ recovery, possible complications or side effects [39,51]. As a result, in practice there is only limited focus on full long term recovery including return to normal activities and return to work [6–7]. Previous studies report that patients often receive conflicting advice about their recovery period after colorectal cancer surgery by health care professionals, and that the degree of guidance and monitoring towards full recovery such as return to work is sometimes limited [44,52]. Furthermore, in general, limited work-related advice is provided by health care professionals [53]. This may be a result of insufficient time at the outpatient clinic or the lack of knowledge of health care professionals about vocational rehabilitation in general [51]. Therefore, often colorectal cancer survivors should decide about the best time to return to work themselves which can be difficult to judge and as such can unnecessarily prolong the time for return to work [12,51]. Previous research on benign gynecological procedures showed that patients achieve earlier return to work if health care professionals provide tailored and personalized advice by eHealth and ICT on the resumption of normal activities including return to work [53–55]. The same goes for a more sustainable work ability which can be achieved when healthcare professionals are more aware of the work-related goals of their patients [39]. Taken together, this evidence suggest that it may be beneficial for colorectal cancer patients and survivors if attention is paid to work related goals during treatment. More collaboration between health care professionals and occupational physicians is receiving increased attention by researchers [56–57], as underlined in a recently performed multicenter randomized controlled trial in which tailored work-related support is provided by an oncological nurse, occupational physician or in a multidisciplinary team [58]. Prognostic factors found in this systematic review can already assist in guiding colorectal cancer patients by health care professionals (especially surgeons and medical oncologists). Four out of six identified prognostic factors are based on the diagnosis or treatment, thus surgeons and oncologists can prepare colorectal cancer patients about the influence of their treatment on the process of return to work.

A recommendation for the absence of uniform definitions for return to work and work disability is to develop an agreed standard “core” set of outcomes that should be used in all trials to facilitate cross-study comparisons, meta-analysis, and minimize outcome reporting bias [59]. Although, there are already three articles available regarding colorectal cancer surgery and core outcome sets [60–62], up to now there is no “core” set. In one of these articles a systematic review regarding patient reported outcomes demonstrated a significant heterogeneity of patient reported outcomes measurement that may hinder comparisons between studies, limit meta-analysis and allow outcome reporting bias. Unfortunately, no core outcome set for full recovery, return to work and return to normal activities is obtained in this set regarding patient reported outcomes [60]. Based on the high heterogeneity of ways that the outcome measures were reported in the studies included in this review, the suggestion to future researchers or developers of core outcome sets is to widen the eligible factors for the core outcome set and to consider to include patient reported outcomes regarding full recovery, return to work and return to normal activities.

In conclusion, *a form of (neo)adjuvant treatment*, *higher age* and *more comorbidities* predispose for later or no return to work for patients recovering from colorectal cancer. *A previous period of unemployment*, *extensive surgery* and *postoperative complications* increase the risk for work disability. Health care professionals need to be aware of these factors to select those patients for intensified rehabilitation to improve return to work and prevent work disability. It is highly recommend to create more uniformity in design and methodology in future studies and there is a need for more high-quality longitudinal studies on this topic.

## Supporting information

S1 File Medline search. (DOCX)

S2 File Prisma checklist. (PDF)

S1 Table Remaining potentially prognostic factors for return to work. * = significant different effect, β = regression coefficient.
(XLSX)

S2 Table Remaining potentially prognostic factors for work disability. * significant different effect.
(XLSX)
