# Supplementary material for: Prognostic factors for return to work and work disability among colorectal cancer survivors; A systematic review
Source: PLoS One. 2018 Aug 15;13(8):e0200720. doi: 10.1371/journal.pone.0200720 (PMC6093640; doi:10.1371/journal.pone.0200720)
Supplement: S1 File — (DOCX) [file pone.0200720.s001.docx]

**Appendix I Medline search**

"Colorectal Neoplasms"[Mesh] OR "Cecal Neoplasms"[Mesh] OR ((colorectal[tiab] OR colon*[tiab] OR rectum[tiab] OR rectal[tiab] OR anus[tiab] OR anal[tiab] OR sigmoid[tiab] OR cecum[tiab] OR cecal[tiab] OR coecum[tiab] OR coecal[tiab] OR caecum[tiab] OR caecal[tiab] OR large bowel[tiab]) AND (cancer[sb] OR neoplasm*[tiab] OR tumor*[tiab] OR tumour*[tiab] OR carcinoma*[tiab] OR cancer*[tiab] OR malignan*[tiab])) AND “Absenteeism"[Mesh] OR "Convalescence"[Mesh] OR "Recovery of Function"[Mesh] OR "Sick Leave"[Mesh] OR "Disability Evaluation"[Mesh] OR "Work Capacity Evaluation"[Mesh] OR "Rehabilitation, Vocational"[Mesh] OR "Return to Work"[Mesh] OR "Sickness Impact Profile"[Mesh] OR "Occupational Health"[Mesh] OR "return to work"[tiab] OR (evaluation*[tiab] AND (disability[tiab] OR work capacity[tiab])) OR "work disability"[tiab] OR "work incapacity"[tiab] OR "work incapability"[tiab] OR "work inhibition"[tiab] OR "working incapacity"[tiab] OR "medical leave"[tiab] OR "sick leave"[tiab] OR "disability leave"[tiab] OR absente*[tiab] OR "work absence"[tiab] OR "disability absence"[tiab] OR convalescen*[tiab] OR sick day*[tiab] OR illness day*[tiab] OR "recovery of function"[tiab] OR "functional recovery"[tiab] OR (recovery[ti] AND function*[ti]) OR "reintegration"[tiab] OR "reemployment"[tiab] OR "job reentry"[tiab] OR "presenteeism"[tiab] OR "sickness absence"[tiab] OR "work absenteeism"[tiab] OR "work day loss"[tiab] OR "work time loss"[tiab] OR "work productivity"[tiab] OR work function*[tiab] OR "work participation"[tiab] OR "work performance"[tiab] OR "performance at work"[tiab] OR "employment status"[tiab] OR "work status"[tiab] OR "occupational health"[tiab] OR disabled person*[tiab] OR disability pension*[tiab] OR job retention*[tiab] OR vocational rehabilitation*[tiab] OR (("Rehabilitation"[Mesh] OR "rehabilitation"[Subheading] OR return*[ti] OR resum*[ti] OR recover*[ti] OR rehabilitat*[ti] OR absen*[tiab]) AND ("Work"[Mesh] OR work[tiab] OR working[tiab]))
